# Development of a loop-mediated isothermal amplification assay for the detection of *Tilletia controversa* based on genome comparison

**DOI:** 10.1038/s41598-021-91098-2

**Published:** 2021-06-02

**Authors:** Somayyeh Sedaghatjoo, Monika K. Forster, Ludwig Niessen, Petr Karlovsky, Berta Killermann, Wolfgang Maier

**Affiliations:** 1 Institute for Epidemiology and Pathogen Diagnostics, Julius Kühn Institute (JKI) – Federal Research Centre for Cultivated Plants, Messeweg 11-12, 38104 Braunschweig, Germany; 2 Institute for Crop Science and Plant Breeding, Bavarian State Research Center for Agriculture, Vöttinger Straße 38, 85354 Freising, Germany; 3 Chair of Technical Microbiology, TUM School of Life Sciences, Technical University of Munich, Gregor-Mendel-Str. 4, 85454 Freising, Germany; 4 Molecular Phytopathology and Mycotoxin Research, University of Goettingen, Grisebachstrasse 6, 37077 Goettingen, Germany

**Keywords:** Agricultural genetics, Fungal pathogenesis

## Abstract

*Tilletia controversa* causing dwarf bunt of wheat is a quarantine pathogen in several countries. Therefore, its specific detection is of great phytosanitary importance. Genomic regions routinely used for phylogenetic inferences lack suitable polymorphisms for the development of species-specific markers. We therefore compared 21 genomes of six *Tilletia* species to identify DNA regions that were unique and conserved in all *T. controversa* isolates and had no or limited homology to other *Tilletia* species. A loop-mediated isothermal amplification (LAMP) assay for *T. controversa* was developed based on one of these DNA regions. The specificity of the assay was verified using 223 fungal samples comprising 43 fungal species including 11 *Tilletia* species, in particular 39 specimens of *T. controversa, *92 of *T. caries* and 40 of *T. laevis*, respectively. The assay specifically amplified genomic DNA of *T. controversa* from pure cultures and teliospores. Only *Tilletia trabutii* generated false positive signals. The detection limit of the LAMP assay was 5 pg of genomic DNA per reaction. A test performance study that included five laboratories in Germany resulted in 100% sensitivity and 97.7% specificity of the assay. Genomic regions, specific to common bunt (*Tilletia caries* and *Tilletia laevis* together) are also provided.

## Introduction

Wheat (*Triticum aestivum*) is the most widely cultivated crop worldwide with a production that reached 734 M tons in 2018^1^ and is still increasing. Several fungal pathogens reduce wheat yield by colonizing different organs of the plant; among them, causal agents of bunt diseases belong to the most important seed- and soil-borne pathogens^2,3^, especially in organic farming. Disease symptoms appear at the heading stage and can be recognized by the formation of black, sooty masses of powdery spores, which replace mostly all grains of a kernel while the modified ovary coat is preserved. The infected grain breaks easily, causing the spread of millions of teliospores.

Common bunt of wheat is caused by *Tilletia caries* and *T. laevis*, dwarf bunt by *T. controversa*, and karnal bunt by *T. indica. Tilletia* belongs to the Exobasidiomycetes within the basidiomycetous smut fungi (Ustilaginomycotina). *Tilletia caries* [syn. *T. tritici*] and *T. laevis* [syn. *T. foetida*] are closely related species present throughout the wheat growing regions of the world^4,5^. Teliospores of common bunt germinate at 15 °C within 1 week even in the absence of light. *Tilletia controversa* causes dwarf bunt and is less widely distributed and restricted to certain regions of the Americas, Europe, and West Asia. For instance, the disease has not been reported from China and Australia. Dwarf bunt is distinguished from common bunt by requiring lower temperature (optimum at 5 °C) and light for the germination of teliospores^6,7^. Germination of *T. controversa* typically takes 3–8 weeks. *Tilletia indica* [syn. *Neovossia indica*] requires temperatures between 15 and 25 °C for germination and takes 2–3 weeks^8^. Karnal bunt is geographically restricted to a few countries namely Afghanistan, India, Iran, Mexico, Nepal, Pakistan, South Africa, Syria, and USA and has not been reported from Europe^9^, where it is treated as an A1 quarantine pathogen by the European and Mediterranean Plant Protection Organization (EPPO)^10^.

Morphology of teliospores and sterile cells comprising their color and size, the size and height of muri, the number of meshes per teliospore diameter, and form of the sori (bunt balls), are traditionally used to distinguish the species of wheat bunt fungi^11^. Differentiation between *T. caries* and *T. controversa* requires extensive experience because of the variability of their teliospores morphology^12^, however *T. laevis*, with its smooth teliospores, generally is easier to distinguish^13,14^. Accurate distinction of dwarf bunt from common bunt and other *Tilletia* species, which are morphologically similar to dwarf bunt, is of high importance. It is required for efficient disease management, as well as for regulatory reasons from a wheat trading perspective. Fifteen countries, including China and Brazil, implemented quarantine measures or restrictions on the number of *T. controversa* teliospores per kernel in their wheat trade^15–17^.

In recent years, several studies have attempted the detection of wheat bunt pathogens using different DNA-based methods. Some of these assays were not intended to differentiate between common and dwarf bunt^18–22^. Assays designed to specifically detect *T. controversa* have been tested only against a limited number of samples^23–27^. Due to the lack of polymorphism in the genomic regions typically used for phylogenetic analyses^18,13,28,29^, alternative DNA regions had to be explored for the development of a species-specific assay. With the advent of new sequencing technologies, it has now become feasible to identify DNA regions for the development of a detection assay without prior knowledge regarding the function of the target sites^30,31^. Here, we employed a comparative genomics approach to detect DNA regions that are conserved in and unique to the *T. controversa* genome. These regions were then used to develop a loop-mediated isothermal amplification (LAMP) assay^32–34^ for the detection of *T. controversa* DNA in pure mycelia and teliospores (from bunt balls). The new assay was validated using a significant number of dwarf and common bunt specimens as well as other wheat pathogens and in an interlaboratory test performance study.

## Results

### Genome comparison and primer design

**Figure 1:**
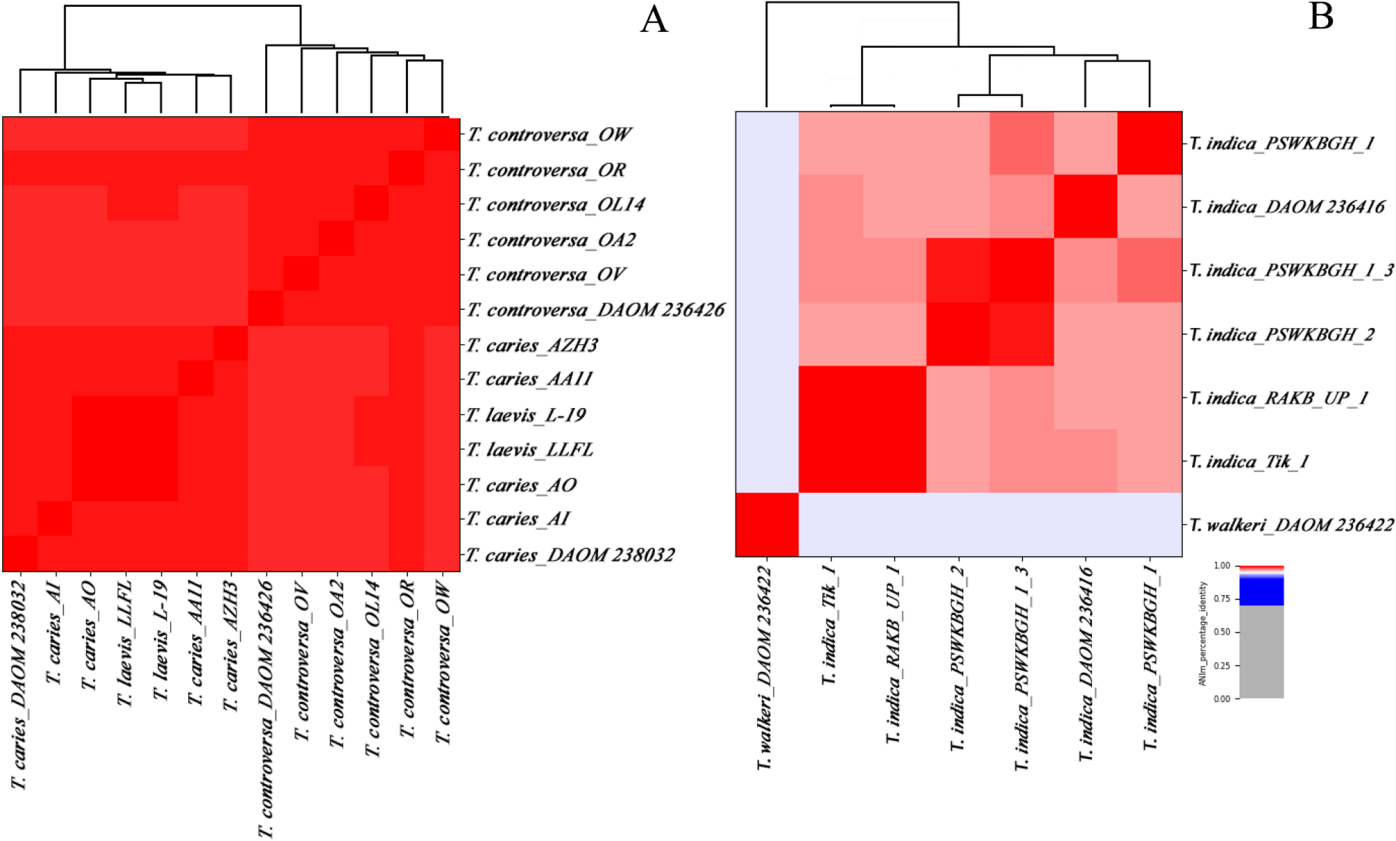
Heatmaps of ANIm percentage identity between genomes of *Tilletia* spp. Pairwise average nucleotide identity between two groups of *Tilletia* species ((**A**) *T. caries*, *T. controversa*, and *T. laevis*, (**B**) *T. indica* and *T. walkeri*) were determined by Pyani and used for the construction of a single linkage dendrogram. The isolates and species assignments are given as row and column labels. The value of the cophenetic correlation coefficient of the hierarchical clustering was 0.97 for (**A**) and 0.99 for (**B**).

Average nucleotide identity (ANI) analysis based on MUMmer^35^ alignment (ANIm) and a single linkage dendrogram were calculated among 20 genomes. For that we divided the genomes into two groups of closely related species, namely *T. caries*, *T. controversa*, and *T. laevis* together, and karnal bunt (*T. indica*) and ryegrass bunt (*T. walkeri*) in another group (Fig. 1A, B and Supplementary Table S1). The alignment coverage is shown in Supplementary Table S2. In general, the higher the alignment coverage and the ANI values, the more identical are the genomes. The genomes of *T. caries*, *T. controversa*, and *T. laevis* (shown in dark red) shared > 99% sequence identity with an average of 91% alignment coverage of the total genome length. In this group, two clades corresponding to common bunt (*T. caries* and *T. laevis*) and dwarf bunt (*T. controversa*) were discriminated using single linkage cluster analysis. *Tilletia caries* and *T. laevis* clustered together in a common clade, with little genetic distance from its neighboring clade comprising the genomes of *T. controversa*. In comparison, the identity within *T. indica* genomes was > 97% with an average of 79% alignment coverage.

The genomes of *T. indica* shared on average 94% sequence identity to the single *T. walkeri* genome. In the second group, *T. indica* samples were separated from the single representative of *T. walkeri.*

The program rapid identification of PCR primers for unique core sequences (RUCS)^36^ was used to identify species-specific sequences in the genome of *T. controversa*. A total of 11,136 unique DNA segments (N50 = 61 bp) were obtained, of which 22 were longer than 1500 bp. These sequences were used for the design of LAMP primers.

A total of 78 primer sets were designed and initially tested for their specificity against a preset comprising eight (three *T. caries*, three *T. controversa*, and two *T. laevis* isolates) selected cultured samples (Supplementary Table S3 shows the list of cultured samples). The primer sets with no false detection and strong amplification as visualized on agarose gels were selected. The second round of testing was performed against the samples of common and dwarf bunt only. The primer sets were excluded when a false positive or false negative reaction occurred. The remaining primer sets were then tested against other *Tilletia* species and fungal pathogens. The primer set with the lowest false detection rate in this round was finally selected. We tested the selected primer set three times independently against the complete sample collection.

**Figure 2:**
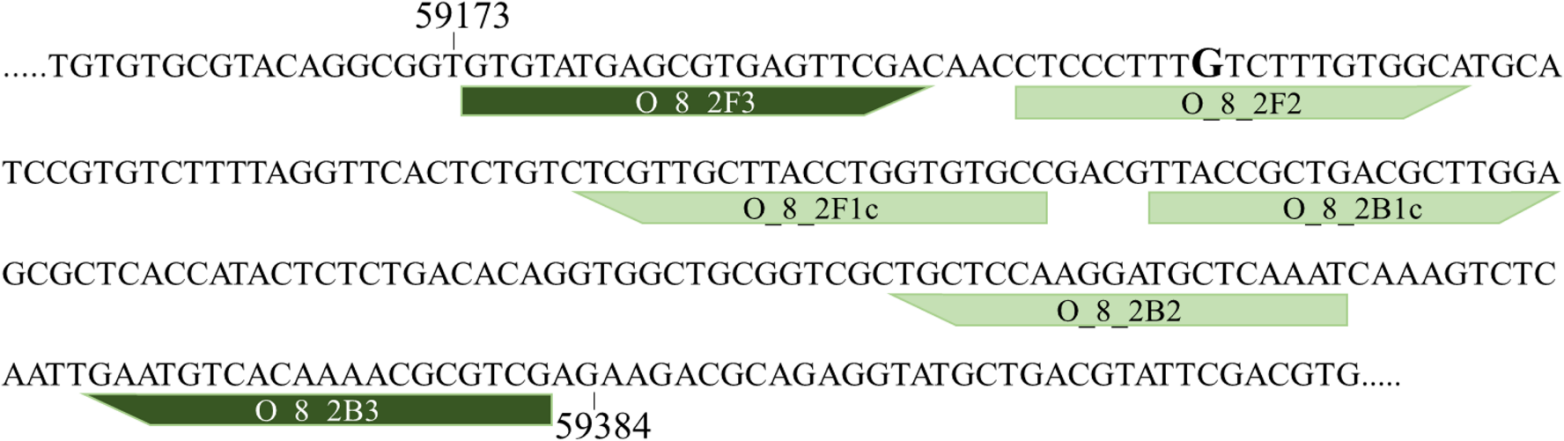
Position and orientation of the primer sequences in the scaffold (accession number CAJHJB010000001) that was used for the development of *T. controversa* LAMP assay. Binding sites for outer primers are shown in dark green, for inner primers in light green. Separation of the binding part is shown with the (_) in the primer sequences. The numbers show the position of nucleotides in the DNA segment. The nucleotide (G) shown in bold is changed to wobble position (K) in the primer sequence, after resequencing of the target region.

Table 1 provides the primer sequences and Fig. 2 shows their location in the target sequence (*T. controversa* isolate OR, scaffold accession number CAJHJB010000001). The primer sequences did not show similarity to any relevant species when blasted against GenBank and this 210 bp intergenic region used for the development of the LAMP assay did not produce a BLAST hit when searched at the DNA level against GenBank’s nucleotide collection.

**Table 1:** Primer sequences used in the LAMP assay.

Primer name	Nucleotide sequences 5′–3′
O_8_2F3	GTGTATGAGCGTGAGTTCGA
O_8_2B3	CGACGCGTTTTGTGACATTC
O_8_2F2	CTCCCTTTKTCTTTGTGGCA
O_8_2B2	ATTTGAGCATCCTTGGAGCA
O_8_2FIP (F1c-F2)	GGCACACCAGGTAAGCAACGA_CTCCCTTTKTCTTTGTGGCA
O_8_2BIP (B1c-B2)	TTACCGCTGACGCTTGGA_ATTTGAGCATCCTTGGAGCA

### Sequence analysis of the species-specific DNA region used for the LAMP assay

**Figure 3:**
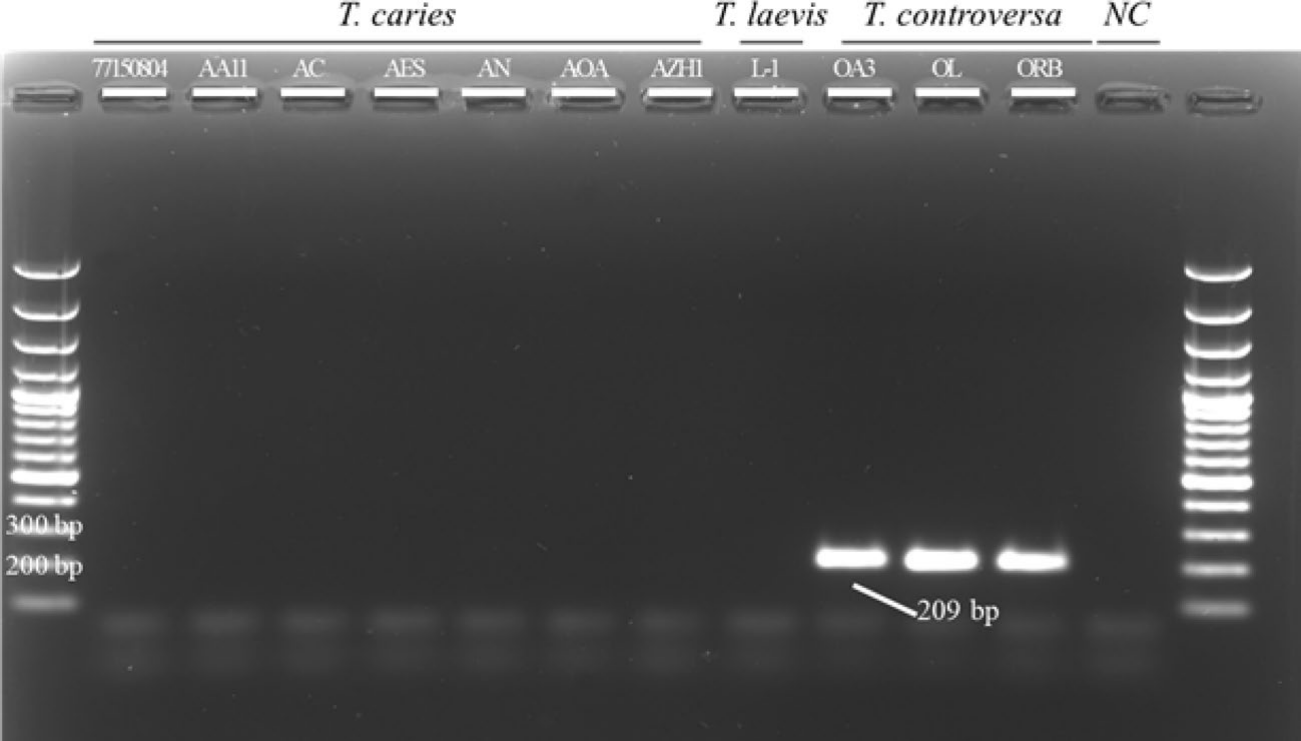
Polymerase chain reaction (PCR) using the outer LAMP primers to amplify the DNA region used for LAMP assay development. PCR products were separated on a 2% agarose gel and visualized using SYBR Safe gel staining. Ladder is a 100 bp Plus GeneRuler and NC is negative control. No amplicon was produced in the absence of *T. controversa* DNA.

PCR products of the predicted length (209 bp) were amplified from DNA of three samples of *T. controversa* from the culture collection (OA3, OR, and ORB isolates) using the LAMP primers O_8_2F3 and O_8_2B3 (Table 1). Sanger-sequencing of the obtained amplicons showed 100% sequence identity with the target DNA region derived from the genome analyses. Only one degenerate nucleotide (K) was introduced into one of the primers (Fig. 2) because at this position a double signal (T/G) was observed in the sequencing chromatogram of an individual isolate. No PCR product was obtained when a subset of DNA obtained from *T. caries* or *T. laevis* samples were used (Fig. 3).

### The LAMP assay and DNA amplification

**Figure 4:**
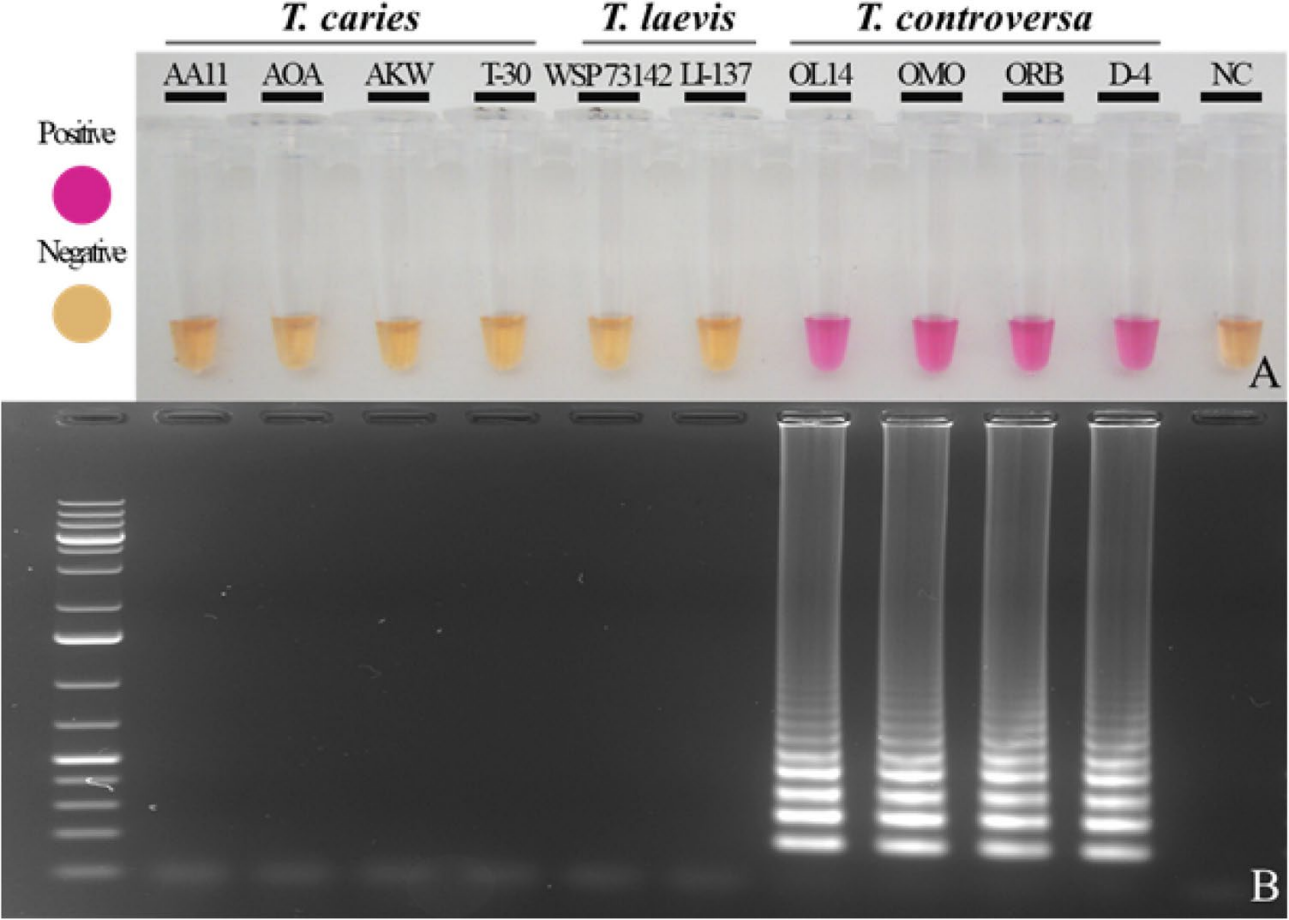
End-point detection of *T. controversa* using neutral red. The LAMP assay was performed at 65 °C for 45 min. (**A**) Colorimetric detection under daylight conditions. Positive reactions appear in pink while negative reactions are light orange. NC is a water control. (**B**) The same reactions separated on a 2% agarose gel and visualized using SYBR safe. A positive LAMP reaction is represented by a ladder-like fragments pattern. NC is the water control and Ladder is 1 kb Plus DNA size marker. The assay detects only *T. controversa* gDNA.

The LAMP assay detected DNA of *T. controversa* obtained from pure fungal cultures as well as from pure teliospores collected from bunt balls. Colorimetric detection of *T. controversa* was achieved by observing a color change of the reaction mixture from orange (no amplification) to pink (positive amplification). Figure 4 shows an example of the colorimetric visualization of the LAMP assay. The products of the reactions were also separated on a 2% agarose gel for confirmation. The typical ladder-like structure of different size amplicons produced in a positive LAMP reaction confirmed that the color change only happened when amplification occurred.

### Verification of LAMP products

**Figure 5:**
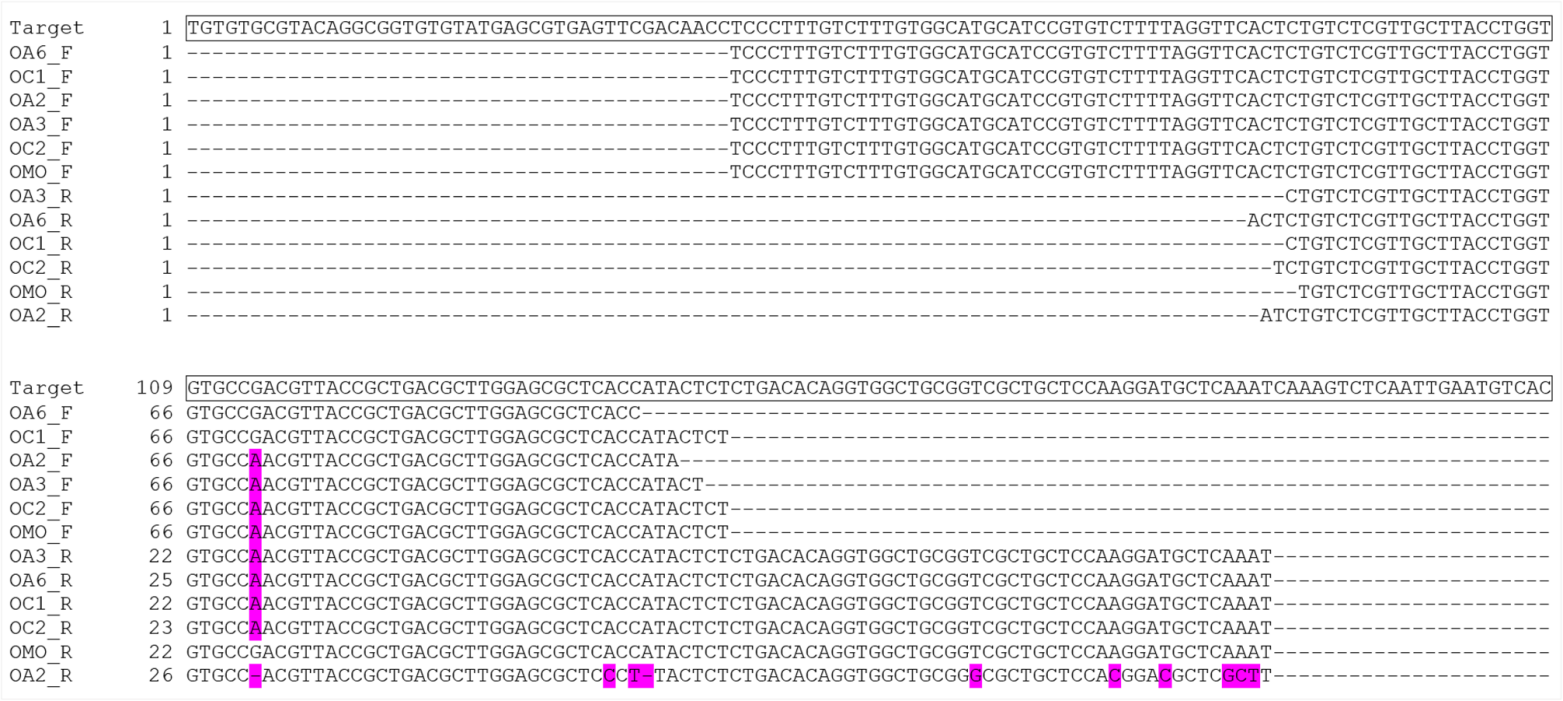
Sequence comparison of the shortest LAMP-product to the target region. The forward and reverse reads obtained from six randomly selected *T. controversa* samples aligned to the target region. The alignment is illustrated using BOXSHADE and non-matching nucleotides to the target region are highlighted in pink.

The amplification of the target DNA segment was confirmed with the DNA of six randomly selected *T. controversa* samples from the culture collection (OA2, OA3, OA6, OC1, OC2, and OMO isolates) as a template in the LAMP assay. Figure 5 shows the multiple alignments of 12 forward and reverse reads obtained from the shortest amplicon against the target DNA region (O_8_2F2 and O_8_2B2 primers). The sequences of the recovered amplicons (151 bp of 152 bp) were almost identical to the target region confirming that the target DNA segment was amplified during the LAMP assay.

### Specificity and limit of detection of the LAMP assay

In total, we tested 223 fungal DNA samples of which 39 belonged to *T. controversa*. Pure cultures were produced for the development of the test. No false positive was observed with 92 *T. caries* and 40 *T. laevis* samples (Supplementary Table S3). Also, no cross-amplification of the assay was observed when testing 40 other fungal phytopathogens including other *Tilletia* species such as *T. cerebrina*, *T. holci*, *T. indica*, *T. lolioli*, *T. menieri*, and *T. olida*. However, *T. trabutii* and a taxonomically uncertainly identified *T. secalis* (GD 1707) were positive under the assay conditions. Wheat did not generate a positive signal when up to 5 ng DNA was used as template.

**Figure 6:**
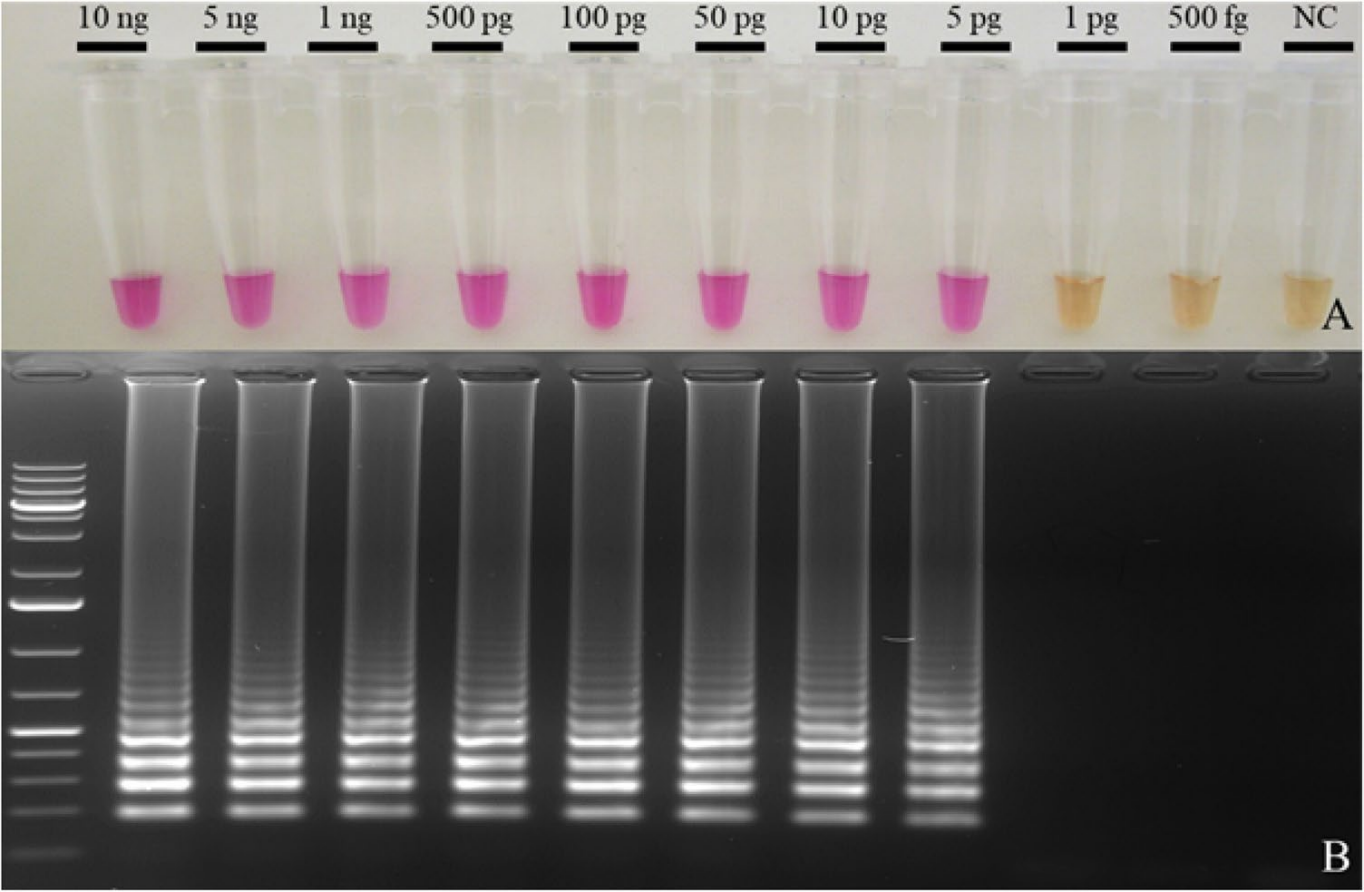
Determination of the LOD of the LAMP assay for *T. controversa*. (**A**) The LAMP assay was carried out with serial dilutions of DNA of *T. controversa* isolate OL and the result was photographed under daylight condition. (**B**) The products were separated on a 2% agarose gel and visualized under UV 360_nm_. NC: water control; Ladder: size marker 1 kb plus.

Figure 6 shows a series of LAMP assays with serial dilutions of pure *T. controversa* DNA. We estimated the LOD as the lowest DNA concentration at which all four repetitions displayed positive results. The assay gave positive results with all replicates at concentrations above 5 pg of DNA per reaction. Three out of four repetitions were amplified when 1 pg of the DNA was tested.

### Reproducibility of the LAMP assay in an interlaboratory test performance

Testing five sets of LAMP test packages prior to sending them to different laboratories showed that all sets detected all *T. controversa* DNA above LOD after 1 week of storage of the reagents at − 20 °C except for betaine, which was stored at + 4 °C. From a total of 80 reactions (five participants testing 15 DNA samples and one negative control each), one false positive (FP), 35 true positives (TP), and 44 true negatives (TN) were reported. The performance parameters are presented in Table 2 and the photos provided by the participants are given in the Supplementary Fig. S1. The positive predictive value (PPV) indicates how many of the test positives are true positives and negative predictive value (NPV) shows how many of the test negatives are true negatives. Both the sensitivity (the fraction of true positive samples that score positive) and the NPV of the assay were 100%. Specificity (the fraction of true negative samples that score negative) and the PPV were 97.7% and 96.5%, respectively.

**Table 2:** Performance parameters of the LAMP assay performance.

Evaluated parameter	Value (%)
True positive fraction (sensitivity)	100
True negative fraction (specificity)	97.7
Positive predicted value (PPV)	96.5
Negative predicted value (NPV)	100

## Discussion

So far, the lack of DNA polymorphisms between the very closely related causal agents of common and dwarf bunt has hampered the development of species-specific DNA-based diagnostic assays for *T. controversa*, as was also shown by other studies^13,28,29,37–39^. However, the recent availability of the whole-genome sequences of *Tilletia* species enabled us to develop a specific LAMP assay.

We calculated > 99% average nucleotide identity (ANI) with a minimum alignment coverage of 88% between common and dwarf bunt if *T. controversa* isolate (OR) is excluded due to its significantly larger assembled genome size. This was in line with ANI values reported by Nguyen et al.^40^, who used independent assemblies of DOAM isolates collection, and our previous study where we analyzed 16 common and dwarf bunt genomes while excluding repetitive regions (Sedaghatjoo et al. under review). The high ANI values indicate a remarkably high genetic similarity of common and dwarf bunt fungi. In comparison, the two closely related species of *T. indica* and *T. walkeri*^41–43^ had ANI values of > 94%. Despite the high similarity between the genomes of common and dwarf bunt fungi, nucleotide polymorphism in the genomes were sufficient to unambiguously separate six *T. controversa* samples from all *T. caries* and *T. laevis*. However, *T. caries* and *T. laevis* could not be differentiated. Interestingly, the ANI values within the sequenced isolates of *T. indica* were lower (> 97%) compared to the ANI values between the three species of *T. caries*, *T. laevis,* and *T. controversa* together (> 99%) indicating a very low genetic diversity between common and dwarf bunt and a relatively high genetic diversity within *T. indica*. Gurjar et al.^44^ also reported high genetic diversity within *T. indica* isolates analyzing single nucleotide polymorphisms (SNP)s and small deletions and insertions (indel)s. Because the percentage of aligned genomic regions between distantly related species was very low, the comparison of all 21 genomes in our ANI analysis was not possible.

The genomic regions of *T. controversa* shared with other *Tilletia* species, but not present in all the *T. controversa* isolates (n = 6) were excluded to be used for the LAMP assay development using RUCS^36^. RUCS employs *k*-mer comparisons to exclude regions shared between target and background genomes. Nguyen et al.^40^ used ten *Tilletia* genomes (one isolate of *T. caries*, two isolates of *T. controversa*, two isolates of *T. laevis*, three isolates of *T. indica*, and two isolates of *T. walkeri*) for the PCR primer design for species-specific detection of *T. controversa*. They limited their sequence comparison to a small number of single-copy protein-coding genes specific and unique to *T. controversa*. The use of RUCS in our study allowed us to include all publicly available sequences and genomes that were not structurally annotated (i.e. genes and their intron–exon locations were not predicted). This approach provided also a higher number of candidate genome regions because it also included intergenic regions. Since the comparison of *k*-mers by RUCS is independent of annotation, it also excludes errors due to annotation ambiguity.

In an attempt to specifically detect *T. caries*, we also searched for conserved and unique regions in their genomes by RUCS (Supplementary Table S4)*.* In *T. caries*, 235 unique and specific regions were found (N50 = 39 bp), the longest spanning 116 bp. In *T. laevis*, we found 228 candidate regions (N50 = 39 bp), the longest of which spanned 215 bp. A minimum length of 200 bp is needed for LAMP because of the optimum distances between primer binding sites. Therefore, differentiation between *T. caries* and *T. laevis* by LAMP appears difficult. Species-specific real-time PCR assays based on the regions identified by RUCS should however be theoretically possible. The number of unique and conserved regions dramatically increased when *T. caries* together with *T. laevis* were treated as a single target. This finding is well in line with the observation that *T. caries* and *T. laevis* could not be differentiated based on the ANI comparisons and clustered together in the single linkage dendrogram. RUCS identified 11,888 regions (N50 = 52 bp) with the longest contig length of 6790 bp, suggesting that developing a common bunt-specific LAMP assay will be feasible.

The updated assembly of four out of 21 genomes (DAOM collection updated to DAOMC) used in this study as well as six additional *Tilletia* genomes has been published by Nguyen et al.^40^ at the time this manuscript was in preparation. We reconfirmed the specificity of the target region identified in this work by comparison of these genomes (See Supplementary Table S5 for the list of accession). The target region used in this study was present in both *T. controversa* genomes and absent from all eight genomes of the other *Tilletia* species. We also repeated the RUCS analysis on all 27 genomes (Supplementary Table S4). The number of extracted species-specific regions dropped sharply, which led to no remaining candidate region for *T. caries*. These results corroborated the pattern observed with 21 genomes. The positions of eleven top-ranked common bunt-specific DNA regions are provided in Supplementary Table S6.

In this study, the LAMP assay for the detection of *T. controversa* was optimized for 45 min at 65 °C using betaine and four salt-free primers with a colorimetric end-point detection. Adding betaine was essential for successful amplification even though the target region was not GC-rich (52.2% GC). This finding is in conflict with a previous report that betaine had no effect on the amplification in non-GC-rich target regions^45^. We suggest that the formation of secondary structures rather than mere GC content may account for the effect of betaine. Increasing the concentration of betaine above 0.5 M did not further improve amplification (data not shown). Here, the LAMP assay was optimized for primers that were not purified by HPLC. Tomita et al.^34^ suggested that HPLC-purification of primers were crucial for a successful LAMP. We compared both HPLC-purified and salt-free primers and found no difference. Although the region in general is long enough to design loop-primers^46^, we did not succeed in integrating them into the assay without compromising the test specificity. Therefore, and because they are generally not essential for the proper functioning of LAMP reactions, we did not include them in the assay. Recently swarm primers have been introduced, which can be added to a LAMP assay in order to improve its general performance^47^. We manually designed and tested a set of swarm primers for the new LAMP assay (data not shown). However, also the addition of these primers neither improved the sensitivity nor the specificity of the assay. Therefore, the final assay comprised only the four basic LAMP primers.

Colorimetric end-point detection of DNA amplification not only shortens the assay time but also reduces the risk of contamination by carryover of LAMP products. Apart from the pH-sensitive indicators neutral red and phenol red^48^, two additional dyes have been widely used for the visualization of LAMP products: hydroxynaphthol blue, added before the reaction^49^ and SYBR Green^34^, added after the completion of the reaction. Using SYBR Green increases the risk of cross-contamination due to the necessity to open the reaction vessels after the LAMP reaction. The color change in hydroxynaphthol blue, which is a metal-sensitive indicator, is occasionally difficult to distinguish^48^. We therefore used neutral red as dye to ensure easy differentiation due to the high contrast between the pink color of a positive reaction and the light orange of a negative reaction.

In this study, the detection limit of the LAMP assay was estimated to be 5 pg total DNA (on average 142 genomes copy when (LOD*6.022 × 10^23^)/(genome length*1 × 10^9^*650)) isolated from pure fungal cultures. This is similar to the sensitivity of the LAMP assay for *T. indica* with the LOD of 10 pg (265 genome copies), reported by Gao et al. and Tan et al.^50,51^. It is however less sensitive than the reported LOD of 1 pg (22 genome copies on average) for the LAMP assay not differentiating among *T. caries*, *T. controversa*, and *T. laevis* published by Pieczul et al.^22^. The rough estimation of detection limits based on genome copy numbers should be taken cautiously since the exact genome sizes of the three species are unknown. The copy number of the target region and loop-primers^46^ may account for these differences. Higher sensitivity in the detection of *T. controversa* by LAMP could presumably be achieved by targeting a multi-copy region. Further investigation is needed to correlate the LAMP detection limit with the number of teliospores per kernel, which is of special interest for seed testing laboratories and farmers, because most of the practiced regulation is based on the number of teliospores per kernel^52^. But irrespective of this, the clear-cut results we obtained by applying LAMP to bunt samples suggest that it might play an important role in the future by differentiating *T. controversa* from *T. caries*, which can be a daunting task given their subtle morphological differences. These differences that can also show some overlaps are especially hard to distinguish if the teliospores are microscoped on filter paper as is done in the official seed testing method.

Broad geographical sampling is crucial for the validation of a species-specific assay, especially when information about the population diversity of the target organism is limited. Additionally, the extensive similarity between the genomes of common and dwarf bunt found here and in other studies^40,53–55^ as well as the probable role of hybridization and recombination between these species^56–58^ in nature makes testing of a geographically broad set of samples essential. One hundred sixty-eight samples of common bunt and dwarf bunt from a variety of geographical locations (Asia, Europe, and North America) were used in this study to evaluate the specificity of the developed LAMP assay using the broadest geographic sampling we could obtain. We made an effort to test both old herbarium samples (collected from 1920 onward) and more recently collected samples to test the independence of the LAMP results from sample age.

A *Tilletia* sample (GD 1707) from *Secale cereale* collected in Germany and identified as *T. secalis* tested positively in the LAMP assay developed in this study. *Tilletia controversa* and *T. secalis* can infect both wheat and rye^4,59,60^ and their differentiation based only on teliospores morphology is not possible^61–63^. Thus, the taxonomic identity of this sample remained ambiguous. Additionally, the assay could not differentiate between *T. controversa* and *T. trabutii*. *Tilletia trabutii* was reported from barley grasses (*Hordeum* spp.) and clustered as the sister group of *T. secalis* in a multilocus phylogenetic analysis^13^. It will be interesting to compare the phylogenetic relationship of *T. secalis* and *T. trabutii* to *T. controversa* on the whole-genome level. Furthermore, *T. controversa,* unlike the majority of smut fungi that are restricted to a single or few closely related host species^64^, has been reported to infect not only wheat but also other members of the *Poaceae* family^65^. We examined *T. controversa* collected from *Elymus repens* and *T. controversa*^11,66,67^ (syn. *T. brevifaciens*^13^) collected from *Thinopyrum intermedium* subsp. *intermedium* (syn *Elymus hispidus*) using our LAMP assay. All were positive. Additionally, *T. bromi* is morphologically similar to *T. controversa* and has similar teliospores germination requirements^14^. These similarities make the distinction of those two species difficult. Although *T. bromi* and *T. controversa* are phylogenetically distinct, they are reproductively compatible under artificial condition^14,68^. We did not have access to any *T. bromi* sample; therefore, the specificity of the LAMP assay toward this species could not be estimated.

The reproducibility of the LAMP assay was also examined in an interlaboratory test performance study including five laboratories, which used different equipments for the amplification. The assay LOD could be successfully reproduced in all the laboratories. We speculate that the most likely reason for one single false-positive result reported was cross-contamination. These results show that the LAMP assay is robust. The assay has potential for several applications in seed testing laboratories, wheat export and import control as well as field applications.

## Methods

### Sample collection and single teliospore cultures

Samples examined in this study are listed in Supplementary Table S3. Host names are listed according to Kew Royal Botanic Gardens online database (https://wcsp.science.kew.org/).

To produce single teliospore cultures, 54 viable samples were randomly selected (marked with * in Supplementary Table S3). Teliospores were surface-sterilized as described by Castlebury et al.^69^. Briefly, bunt balls were crushed using a pair of sterile fine-point forceps and wheat tissue was carefully removed. The teliospores were immersed in 0.26% v/v NaClO for 30 s, pelleted by centrifugation in a benchtop microcentrifuge for 10 s and rinsed twice with sterile, distilled water. Surface-sterilized teliospores were streaked on 1.5% water-agar and incubated either at 5 °C under constant light (*T. controversa* teliospores) or at 15 °C in darkness (*T. caries* and *T. laevis*). A single germinated teliospore of each sample was then transferred to M-19 agar medium^70^ using a sterile needle and incubated at 15 °C in the dark. Medium was supplemented with penicillin G (240 mg/L) and streptomycin sulfate (200 mg/L). The developing mycelium was scraped from the medium using a flat blunt spatula, freeze-dried at − 40 °C for 48 h and kept at + 4 °C until use. Cultures of other fungal species were grown on PDA medium and stored at + 4 °C.

### Extraction of DNA from fungal mycelia and spores

Total DNA (gDNA) including mitochondrial DNA and mycoviruses was isolated from both mycelia and spores (cultured isolates) or only from spores (uncultured samples). For extraction of gDNA from fungal cultures, 10–30 mg of lyophilized mycelium were homogenized by shaking with four sterile tungsten carbide beads of 4 mm diameter in 2 ml reaction tubes at 22 Hz for 50 s using a tissue lyser. The bead beating step was repeated once. The tubes were shaken vigorously between the disruption steps to loosen mycelium from the bottom of the tubes.

For gDNA extraction directly from spores, 10–25 mg spores were surface-sterilized as described above and rinsing water was carefully removed. Four 1 mm and four 4 mm sterile tungsten carbide beads were added to each reaction tube. The tubes were then frozen in liquid nitrogen and the spores were disrupted in the tissue lyser at 22 Hz for 50 s. The procedure including cooling the samples in liquid nitrogen was repeated twice. After tissue disruption, DNA was extracted using the Qiagen DNeasy Plant Mini kit (Qiagen GmbH, Hilden, Germany) according to the manufacturer’s protocol.

### Genome comparisons and identification of DNA segments specific to *T. controversa*

Twenty one genome sequences used in this study are listed in Table 3.

**Table 3:** Genome sequences used in this study and their accession numbers.

No	Species	Isolate (voucher number)	Assembly accession numbers	Genome size (Mbase)	Reference
1	*T. caries*	AA11 (CBS 144825)	GCA_905072865.1	31.51	Sedaghatjoo et al. under review
2	*T. caries*	AI (CBS 145171)	GCA_905068135.1	31.84	Sedaghatjoo et al. under review
3	*T. caries*	AO (CBS 145172)	GCA_905071735.1	30.46	Sedaghatjoo et al. under review
4	*T. caries*	AZH3 (CBS 145166)	GCA_905071745.1	31.38	Sedaghatjoo et al. under review
5	*T. caries*	DAOM 238032	GCA_001645005.1	29.54	NA
6	*T. controversa*	DAOM 236426	GCA_001645045.1	28.84	NA
7	*T. controversa*	OA2 (CBS 145169)	GCA_905071725.1	32.05	Sedaghatjoo et al. under review
8	*T. controversa*	OL14 (CBS 145167)	GCA_905071785.1	30.83	Sedaghatjoo et al. under review
9	*T. controversa*	OR (CBS 144827)	GCA_905071765.1	49.87	Sedaghatjoo et al. under review
10	*T. controversa*	OV (CBS 145170)	GCA_905071775.1	29.54	Sedaghatjoo et al. under review
11	*T. controversa*	OW (CBS 145168)	GCA_905071705.1	31.24	Sedaghatjoo et al. under review
12	*T. horrida*	QB-1	GCA_001006505.1	20.10	Wang et al.^71^
13	*T. indica*	DAOM 236416	GCA_001645015.1	30.38	NA
14	*T. indica*	PSWKBGD_1_3	GCA_001689965.1	43.73	NA
15	*T. indica*	PSWKBGH_1	GCA_001689995.1	37.46	Sharma et al.^72^
16	*T. indica*	PSWKBGH_2	GCA_001689945.1	37.21	Sharma et al.^72^
17	*T. indica*	RAKB_UP_1	GCA_002220835.1	33.77	Gurjar et al.^73^
18	*T. indica*	Tik_1	GCA_002997305.1	31.83	Kumar et al.^74,75^
19	*T. laevis*	L-19 (CBS 145173)	GCA_905071715.1	31.00	Sedaghatjoo et al. under review
20	*T. laevis*	LLFL (CBS 144826)	GCA_905071755.1	30.98	Sedaghatjoo et al. under review
21	*T. walkeri*	DAOM 236422	GCA_001645055.1	24.34	NA

Average nucleotide identity (ANI), the alignment coverage between genomes, and hierarchical clustering were calculated and reconstructed using Pyani (v 0.2.10)^76^ which employs MUMmer (ANIm mode) to align genomes, with default parameters (-m ANIm -g). The cophenetic correlation coefficient of the hierarchical clustering was calculated in RStudio (Version 1.1.463)^77^.

Conserved and unique DNA regions of *T. controversa* were extracted using RUCS (rapid identification of PCR primers for unique core sequences) v. 1.0^36^ (https://cge.cbs.dtu.dk/services/RUCS/) with default parameters. The six target genomes (*T. controversa*) were defined and grouped as positive while the remaining 15 *Tilletia* genomes (*T. caries*, *T. horrida*, *T. indica*, *T. laevis*, and *T. walkeri*) were defined and grouped as negative data set or exclusion criteria. Extracted contigs found in the unique-core-sequences-contigs output file of RUCS that were longer than 1500 bp were selected as targets for LAMP development. To check these contigs for similarities with nontarget genomes, a custom BLAST database of all available *Tilletia* genomes (n = 15) excluding *T. controversa* genomes was constructed.

### Primer design for the LAMP assay

A set of two inner and two outer primers were designed for the unique DNA contigs to *T. controversa* by PrimerExplorer V5 (http://primerexplorer.jp) with default parameters. Since PrimerExplorer does not accept sequences longer than 2000 bp, we split sequences exceeding this limit. The designed primers were subjected to MegaBLAST against the non-redundant database ‘nr/nt’ of the National Centre for Biotechnology Information (NCBI) to examine their similarity with other relevant species.

### Sequence analysis of DNA segment used for the LAMP assay

To confirm the nucleotide sequence of the target region obtained by RUCS, we used the outer primers (F3 and B3) of LAMP as forward and reverse primers, respectively, in a conventional PCR and sequenced the obtained PCR product for a subset of samples. The PCR was conducted in 50 μL reaction mixtures containing 5 μL of 10 × DreamTaq (Thermo Scientific, Vilnius, Lithuania), 0.2 mM of each of the four deoxynucleotide triphosphates (dNTPs, Thermo Scientific), 0.2 µM concentration of each forward and reverse primers, 1.25 U of *Taq* DNA polymerase (DreamTaq DNA polymerase, Thermo Scientific, Vilnius, Lithuania), and 1 μL of DNA template (5 ng/µL). Initial denaturation was conducted at 95 °C for 2 min, followed by 35 cycles of denaturation at 95 °C for 30 s, annealing at 60 °C for 30 s and extension at 72 °C for 60 s. The final extension was performed at 72 °C for 10 min in a thermal cycler. Following PCR, 5 µl per reaction combined with 2 µL of 6 × loading buffer (Thermo Scientific, Vilnius, Lithuania) were loaded onto a 2% agarose gel (w/v). The electrophoresis was run at 8 V cm^−1^ for 45 min in 1 × TAE buffer^78^. PCR fragments were stained using SYBR^®^ Safe DNA gel stain. The gel was visualized under UV 360_nm_ using a digital imaging system. PCR products were purified using the DNA Clean & Concentrator™-5 kit (Zymo Research, Irvine, California, USA) according to the manufacturer’s instructions. Purified PCR products were Sanger-sequenced (Eurofins Genomics GmbH, Ebersberg, Germany) from both ends using PCR primers.

### LAMP assay and verification of the LAMP products

The LAMP master mix (25 μL) contained 2.5 µL 10 × amplification buffer (100 mM KCl, 100 mM (NH_4_)_2_SO_4_, pH 8.7), 2 μM of each inner primer and 0.2 μM for each outer primer (all salt-free), 8 mM MgSO_4_, 1.4 mM concentration of each of the four deoxynucleoside triphosphates (dNTPs, Thermo Fisher), 0.5 M betaine (Sigma-Aldrich, Darmstadt, Germany), 8 U *Bst* DNA Polymerase 2.0 (New England Biolabs, Frankfurt, Germany), and 100 µM neutral red (Sigma-Aldrich, Darmstadt, Germany) prepared according to Niessen et al.^79^. One µL of DNA template was added per reaction. The reaction mixture was incubated at 65 °C for 45 min in a thermal cycler. The reaction was terminated by heating to 80 °C for 5 min. The tubes were photographed with a digital camera under daylight conditions. Gel electrophoresis of the LAMP products was performed as described above but the separation lasted 120 min. Either a 100 bp plus or 1 kb Plus GeneRuler (Thermo Scientific, Vilnius, Lithuania) were used as DNA size markers in all electrophoretic gels.

To confirm that the amplification corresponded to the target DNA region, the shortest amplicon of six positive LAMP reactions was excised from a 2% agarose gel (w/v) (described previously) and recovered using the Zymoclean™ Gel DNA Recovery Kit (Zymo Research, Irvine, USA) according to the manufacturer’s instructions. The recovered amplicons were Sanger-sequenced using primers F2 and B2. Consensus sequences of all forward and reverse reads produced and trimmed using Sequencher™ 5.4.6 (Gene Codes Corporation, Ann Arbor, Michigan, USA) were pairwise aligned to the sequence of the target DNA region.

### Determination of the specificity and limit of detection of the LAMP assay

The specificity of the LAMP assay was determined by applying the assay to DNA extracted from *Tilletia* cultures and teliospores, and from a range of phylogenetically distant fungal pathogens (Supplementary Table S3) under the described LAMP conditions. The following groups of pathogens were selected as negative controls: (1) closely related species with a “sister group” phylogenetic relationship to the target pathogen (92 *T. caries* and 40 *T. laevis* samples); (2) further pathogens related to the target species (e.g. eight other *Tilletia* spp. and species of Ustilaginomycetes); (3) common wheat pathogens (e.g. *Fusarium* spp.); (4) fungi that are abundant in the environment due to their strong sporulation and airborne mode of distribution (e.g. *Penicillium* spp*.*). In addition, wheat DNA extracted from seedlings grown under sterile conditions was tested.

Limit of detection (LOD) was defined as the lowest amount of analyte detectable in a single reaction^80^. DNA obtained from a pure culture of *T. controversa* isolate OL was quantified using Qubit^®^ 3.0 Fluorometer (Thermo Fisher Scientific, Darmstadt, Germany) and used to prepare a dilution series at the concentrations of 10,000, 5000, 1000, 500, 100, 50, 10, 5, 1, and 0.5 pg per assay.

### Reproducibility of the LAMP assay in a test performance study

To evaluate the reproducibility and specificity of the LAMP assay, we conducted a test performance study with five German participants including plant protection agencies and seed testing laboratories. Total DNA of four *T. caries*, five *T. controversa*, and three *T. laevis* sample was extracted from teliospores as described above. The concentration of DNA stocks was determined using a Qubit^®^ 3.0 Fluorometer and adjusted to 500 pg/µL. One of the *T. controversa* sample was additionally prepared in a serial dilution of 50, 5, and 0.5 pg/µL. All 15 DNA samples were coded, and aliquots were dispatched to the participating laboratories. Several batches of the LAMP master mix were prepared independently and assigned randomly to the participating laboratories. Homogeneity and stability testing were performed under described conditions with five randomly selected batches. The assays were performed on different days shortly before the chemicals and samples were distributed by an express delivery service while kept at − 20 °C (except for betaine). The participants were asked to provide a photo of the reaction tubes and assign the samples as positive or negative according to the color of the reaction mixture after performing the assay. The results were evaluated according to Hajian-Tilaki^81^ using performance parameters shown in Table 4.

**Table 4:** Evaluation of the LAMP assay.

Performance parameters	Calculation
True positive fraction (sensitivity)	True positive (TP)/(TP + false negative (FN))
True negative fraction (specificity)	True negative (TN)/(TN + false positive (FP))
Positive predicted value (PPV)	TP/(TP + FP)
Negative predicted value (NPV)	TN/(TN + FN)

## Supplementary Information

Supplementary Information 1.

Supplementary Information 2.

## Data Availability

The datasets analyzed during the current study are publicly available as mentioned in the text.
