# Supplementary material for: Development of a loop-mediated isothermal amplification assay for the detection of Tilletia controversa based on genome comparison
Source: Sci Rep. 2021 Jun 2;11:11611. doi: 10.1038/s41598-021-91098-2 (PMC8172862; doi:10.1038/s41598-021-91098-2)
Supplement: Supplementary file 1 — Supplementary Information 1. [file 41598_2021_91098_MOESM1_ESM.docx]

**Development of a loop-mediated isothermal amplification assay for the detection of *Tilletia controversa* based on genome comparison**

Somayyeh Sedaghatjoo^1*^, Monika K. Forster^2^, Ludwig Niessen^3^, Petr Karlovsky^4^, Berta Killermann^2^, Wolfgang Maier^1^

^1^ Julius Kühn Institute (JKI) – Federal Research Centre for Cultivated Plants, Institute for Epidemiology and Pathogen Diagnostics, Messeweg 11-12, 38104 Braunschweig, Germany

^2^ Institute for Crop Science and Plant Breeding, Bavarian State Research Center for Agriculture, Vöttinger Straße 38, 85354 Freising, Germany

^3^ Chair of Technical Microbiology, TUM School of Life Sciences, Technical University of Munich, Gregor-Mendel-Str. 4, 85454 Freising, Germany

^4^ Molecular Phytopathology and Mycotoxin Research, University of Goettingen, Grisebachstrasse 6, 37077 Goettingen, Germany

*Email of the corresponding author: s.sedaghatjoo@gmail.com


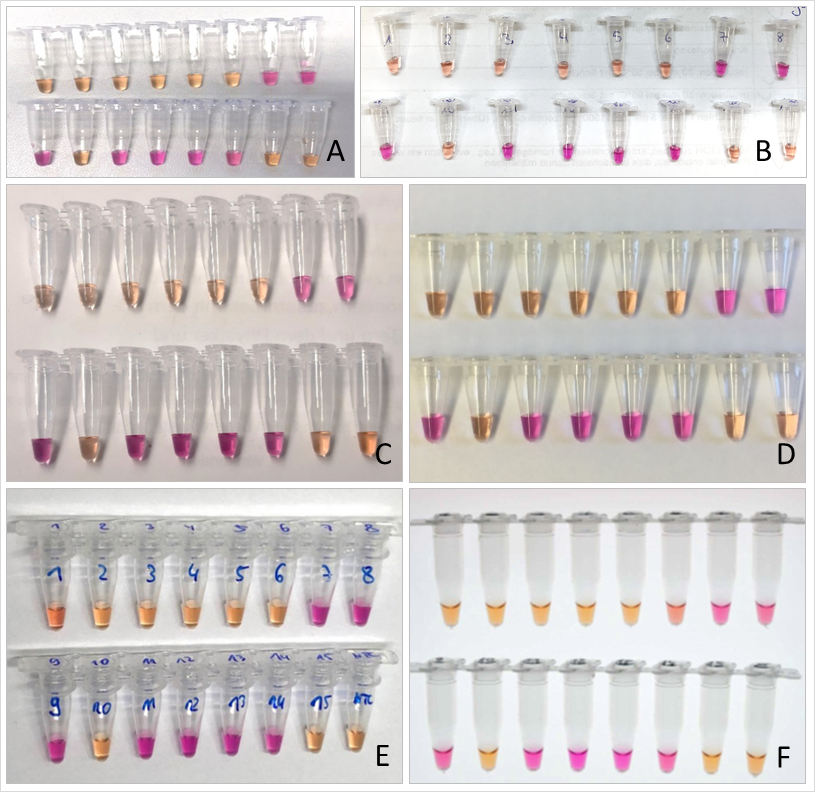


Supplementary Figure S1 Photos of the interlaboratory test performance study of the developed LAMP assay. A: the reference photo taken by the organizer. B – F: photos that are received from the participants. The reaction color changes to pink when *T. controversa* is detected. The False Positive (FP) reported reaction is marked by (*). Photo F is color intensified by a photo-editing software (Adobe Photoshop version 6.0). The sensitivity and specificity of the test were 100 and 97.7%, respectively.

*
